# Comparison of Reverse Transcriptase (RT) Activities of Various M-MuLV RTs for RT-LAMP Assays

**DOI:** 10.3390/biology11121809

**Published:** 2022-12-13

**Authors:** Igor P. Oscorbin, Lidiya M. Novikova, Maxim L. Filipenko

**Affiliations:** Institute of Chemical Biology and Fundamental Medicine, Siberian Branch of the Russian Academy of Sciences (ICBFM SB RAS), 8 Lavrentiev Avenue, 630090 Novosibirsk, Russia

**Keywords:** RT-LAMP, LAMP, isothermal amplification, reverse transcriptase, superscript, M-MuLV RT, Sto7d, fusion protein

## Abstract

**Simple Summary:**

Reverse transcriptases (RTs) are a family of enzymes synthesizing DNA with RNA as a template, and are used in almost all studies related to RNA. M-MuLV RT is widely used in diagnostics methods, including in reverse-transcription loop-mediated isothermal amplification (RT-LAMP). The performance of various reverse transcriptases in RT-LAMP remains poorly studied. Here, we report the first direct comparison of various M-MuLV RTs in RT-LAMP. The enzymes studied contained different numbers of mutations or an additional Sto7d protein. Several parameters were assessed: optimal reaction temperature, enzyme concentration, reverse transcription time, a minimal amount of RNA template, and tolerance to inhibitors. Mutations increased the optimal temperature of the reverse transcription up to 5–10 °C. All of the RTs were suitable for RT-LAMP with RNA templates in the range of 10^1^–10^6^ copies per reaction. Highly mutated enzymes were more tolerant to most of the inhibitors, but more sensitive to high concentrations of NaCl. The results presented could help select the optimal enzyme for novel LAMP-based diagnostic tests.

**Abstract:**

Reverse transcriptases (RTs) are a family of enzymes synthesizing DNA using RNA as a template and serving as indispensable tools in studies related to RNA. M-MuLV RT and its analogs are the most commonly used RTs. RTs are widely applied in various diagnostics methods, including reverse-transcription loop-mediated isothermal amplification (RT-LAMP). However, the performance of different RTs in LAMP remains relatively unknown. Here, we report on the first direct comparison of various M-MuLV RTs in RT-LAMP, including enzymes with a different number of mutations and fusions with Sto7d. Several parameters were assessed, namely: optimal reaction temperature, enzyme concentration, reverse transcription time, a minimal amount of RNA template, and tolerance to inhibitors. Mutations increased the optimal reaction temperature from 55 °C to 60–65 °C. All of the RTs were suitable for RT-LAMP with RNA templates in the range of 10^1^–10^6^ copies per reaction. Highly mutated enzymes were 1.5–3-fold more tolerant to whole blood, blood plasma, and guanidinium, but they were two-fold more sensitive to high concentrations of NaCl. The comparison of different RTs presented here could be helpful for selecting the optimal enzyme when developing novel LAMP-based diagnostic tests.

## 1. Introduction

Reverse transcriptases (RTs) are a family of enzymes utilizing RNA as a template to synthesize DNA and participating in various processes such as retrovirus propagation and telomere end elongation [1,2]. To date, RTs have remained a cornerstone of almost all studies related to RNA. These enzymes synthesize more stable complementary DNA (cDNA), which is more suitable for downstream analysis. Since the discovery of RTs in 1970 [3,4], numerous RTs have been identified and biochemically characterized [5,6,7,8]. Most of these enzymes were found in retroviruses. Currently, the most popular RTs are M-MuLV RT (from a Moloney Murine Leukemia Virus) and its various mutant analogs. Unlike other retroviral RTs, M-MuLV RT is active as a single-subunit enzyme [9] and has been extensively studied for several decades [9,10,11,12,13,14,15]. The main goal in generating M-MuLV RT mutants is to achieve a higher optimal temperature and thermal stability [16,17,18]. By improving these parameters, researchers intend to increase the efficacy of cDNA synthesis on RNA templates with a complex secondary structure [11,16]. The thermal stability of M-MuLV RT mutants is directly proportional to the enzyme’s affinity towards a primer–template complex [11,17,19]. Mutations in template-interacting regions or fusion with additional DNA-binding protein domains could result in tether binding to the template [16,17,18,20,21]. Fortunately, thermostable M-MuLV RT mutants also demonstrated a higher tolerance to common amplification inhibitors, including heparin, formamide, NaCl, and humic acids [20,22]. The efficacy of RT-PCR with various improved M-MuLV RTs was also higher than with the native enzyme.

Loop-mediated isothermal amplification (LAMP) is a powerful technique for DNA and RNA detection under isothermal conditions [23]. LAMP provides a promising alternative to conventional PCR, with cheaper equipment (no usage of thermocyclers), equal analytical specificity, sensitivity, and increased robustness to amplification inhibitors [24,25]. LAMP has become the basis of testing for multiple pathogens, including influenza virus, Zika virus, malaria parasite, *Mycobacterium tuberculosis*, and SARS-CoV-2 [26,27,28,29,30]. For RNA testing, LAMP could be performed after reverse transcription in a separate tube or coupled with reverse transcription in a single tube format (RT-LAMP). Since the beginning of the current SARS-CoV-2 pandemic, the interest of clinicians in RT-LAMP has rapidly grown [25]. Plenty of test systems based on RT-LAMP were designed for SARS-CoV-2 diagnostics, mostly for point-of-care testing [25,31,32,33,34,35,36]. The latter assumes diagnostics at the bedside without sophisticated sample preparation and DNA or RNA purification. Thus, optimizing RT-LAMP conditions, including selecting the most effective RT, can be crucial, given that RTs have various tolerance levels to inhibitors and optimal reaction conditions. 

Commercial vendors offer several RTs suitable for RT-LAMP, with multiple studies reporting a decent RT-LAMP efficacy with different RTs [32,35,37,38,39,40]. Despite a relatively long study history, information about the performance of various RTs in LAMP remains scarce and insufficient. Thus, a direct comparison of RTs using RT-LAMP could facilitate the choice of the most suitable enzyme. The present study partially fills that gap and focuses on the efficacy and inhibitor tolerance of Superscript III, Superscript IV, M-MuLV RT without RNAse H- domain, and M-MuLV RT with fused Sto7d protein.

## 2. Materials and Methods

### 2.1. Standard Plasmids with Genome Fragments of SARS-CoV-2 and MS2 Phage

First, 200 nt DNA fragments of the SARS-CoV-2 E gene complementary to the SARS-CoV-2 RNA (GenBank ID NC_045512.2) were obtained by PCR from separate oligonucleotides using the Polymerase Cycling Assembly (PCA) method [41]. Oligonucleotides for the assembly of SARS-CoV-2 viral DNA fragments were chosen by the GeneCut algorithm (Unipro, ICBFM SB RAS, Novosibirsk, Russia) and synthesized on an automatic DNA/RNA synthesizer ASM-2000 (Biosset, Novosibirsk, Russia).

The DNA fragment obtained and the pBlueScript II SK (+) vector were hydrolyzed by restriction endonucleases EcoRI and BamHI (SibEnzyme, Novosibirsk, Russia) and ligated using 100 units of T4 DNA ligase (Biosan, Novosibirsk, Russia). A ligase mixture was then used to transform the competent cells of the XL1-Blue *E. coli* strain (Stratagene, La Jolla, CA, USA). The structure of plasmid clones was proved by Sanger sequencing performed on ABI 3130XL GeneticAnalyzer (Applied Biosystems, Bedford, MA, USA), using BigDye 3.1 kit (Genomics Core Facility, ICBFM SB RAS, Novosibirsk, Russia). The pBlueScript II SK (+) containing the MS2 phage genome was synthesized artificially (Shanghai RealGene Bio-tech, Shanghai, China). Recombinant plasmid DNA pBlueScript-CoV-2 and pBlueScript-MS2 were isolated from 50 mL of night cultures in LB medium using the QIAGEN Plasmid Midi Kit (Qiagen, Venlo, The Netherlands), in accordance with the manufacturer’s protocol.

The concentration of standard plasmid DNA obtained was measured using the Qubit™ BR kit (Invitrogen, Waltham, MA, USA). Then, 2 µg of each plasmid DNA was linearized by BamHI restriction endonuclease. The resulting linearized plasmid standards were diluted to a concentration of 10^5^ and further down to two copies of plasmid DNA per µL in a sterile buffer containing 10 mM Tris-HCl (pH 7.6) and 5 ng/mL yeast RNA.

### 2.2. In Vitro RNA Synthesis

Linearized by HindIII, pBlueScript-CoV-2 served as a template for in vitro RNA synthesis. The reactions were carried out in a total reaction volume of 50 µL, containing 2 µg of DNA template, 1 mM of each NTP, 100 units of T7 RNA polymerase (SibEnzyme, Novosibirsk, Russia), and 1x reaction buffer (50 mM Tris-HCl, pH 7.5, 6 mM MgCl_2_, 10 mM DTT, 2 mM spermidine). After 2 h of incubation at 37 °C, 100 units of DNase I (Worthington Biochemical, Lakewood, NJ, USA) were added to the reaction mixtures, followed by incubation for 15 min at 37 °C. Synthesized RNA fragments were isolated by phenol–chloroform extraction and precipitated with isopropanol [42]. Purified RNAs were dissolved in DEPC-treated water and stored at −80 °C.

### 2.3. Isolation of MS2 Phage RNA

The MS2 phage was grown using the modified protocol of Sambrook and Russel [43]. The fresh night culture of the *E. coli* K12 strain was diluted in 3 mL of MS2 medium to OD_600_ = 1 (equal to 1 × 10^9^ cells/mL), followed by the addition of the MS2 phage to reach a phage/cell ratio of 5. The cultures were incubated at 37 °C for 20 min, mixed with 500 mL of preheated MS2 medium, and incubated under the same conditions for 12 h. The chloroform was added to lyse the cells, and the culture was vortexed for 10 min at 37 °C. The lysate was treated by DNase I and RNase A (50 mg/mL each) for 30 min at 37 °C. Then, NaCl was added to a final concentration of 1 M. The mixture was incubated on ice for 1 h, with the debris separated by centrifugation (10 min, 11,000× *g*) at 4 °C. The supernatant was supplied by an additional amount of ammonium sulfate to reach a final concentration of 50% (m/m), the mixture was incubated for 2 h at 4 °C, and the phage particles were precipitated by centrifugation (30 min, 11,000× *g*) at 4 °C. The precipitated phage particles were dissolved in 30 mL of the TSM buffer (20 mM Tris-HCl, pH 7.4, 150 mM NaCl, 2 mM CaCl_2_, 2 mM MgCl_2_). MS2 RNA was isolated from the phage particles using QIAamp Circulating Nucleic Acid Kit (Qiagen, Venlo, Netherlands) according to the manufacturer’s protocol and stored at −80 °C.

### 2.4. Expression and Purification of Reverse Transcriptases

The cloning of chimeric and mutant RTs was described in our previous work [20]. In this study, several enzymes were examined, namely: M-MuLV RT with Sto7d protein from *Sulfolobus tokodaii* at C-terminus, M-MuLV RT with previously characterized D200N, T330P, L139P mutations [16], M-MuLV RT with D200N, T330P, L139P, and Sto7d protein at the C-terminus. For the RTS sequences, refer to the Supplementary Materials. All studied enzymes are listed in Table 1.

The BL21 (DE3) pLysS (Promega, Madison, WI, USA) strain of *E. coli* cells harbouring the plasmid encoding RT was grown to OD_600_ = 0.3 in LB medium at 37 °C. Four litres of LB in a LiFlus GX fermenter (Biotron Inc., Bucheon, South Korea) was inoculated with 40 mL of the starter culture, and the cells were grown to OD_600_ = 0.6 at 37 °C. The expression was induced by adding IPTG up to 1 mM concentration. After induction for 4 h at 37 °C, the cells were harvested by centrifugation at 4000× *g* and stored at −70 °C.

For protein purification, the cell pellets were resuspended in a lysis buffer (50 mM Tris-HCl pH 7.0, 2.5 mM MgCl_2_, 0.1 mM CaCl_2_, 1 mM PMSF, 1 mg/mL lysozyme) and incubated for a 30 min on ice, followed by sonication. The lysates were treated for 15 min at 37 °C with DNAse I (1 µg/mL) for DNA digestion, followed by centrifugation at 14,000× *g*. The resulting supernatants were loaded onto a 5 mL IMAC column (Bio-Rad, CA, Hercules, USA) pre-equilibrated with buffer A (50 mM Tris-HCl pH 7.0, 0.3 M NaCl), followed by washing the column with 25 mL of buffer A with 1 M of NaCl. The bound proteins were eluted using buffer B (buffer A with 0.3 mM imidazole). After affinity chromatography, the fractions with RTs were pooled and loaded onto a 2 mL Macro-Prep DEAE Resin (Bio-Rad, column CA, Hercules, USA) pre-equilibrated with buffer C (50 mM Tris-HCl, 0.1 mM EDTA, pH 7.5). The column was washed with 10 mL of buffer C, and the bound proteins were eluted with a 0–100% linear gradient of buffer D (50 mM Tris-HCl, 1 M NaCl, 0.1 mM EDTA, pH 7.5). The fractions with RTs were pooled, dialyzed against the storage buffer (50 mM Tris-HCl, 150 mM NaCl, 0.1 mM EDTA, 50% Glycerol, 0.1% NP-40, pH 7.5), and stored at −20 °C. All of the fractions from each step were analysed by SDS-PAGE. The purity of the preparations was not less than 95%. The concentration of purified proteins was measured using a standard Bradford assay.

### 2.5. RNA-Dependent DNA-Polymerase Activity Measurement

The specific activity of the RT was assayed by using radiolabelled nucleotide incorporation. The reaction mix (50 µL) contained 0.4 mM poly(rA)/oligo(dT)25 (concentration defined by oligo(dT)25), 0.5 mM α-[32P]-dTTP (4 Bq/pmol), 50 mM Tris-HCI (pH 8.3), 6 mM MgCl_2_, and 10 mM DTT. The reactions were initiated by adding the enzyme on ice, with the samples immediately transferred to a preheated thermal cycler for incubation at 37 °C for 10 min, followed by inactivation by heating at 90 °C for a 1 min. The reaction products were collected on DE81 paper (Sigma-Aldrich, St. Louis, MO, USA), washed twice with 0.5 M Na_2_HPO_4_, and counted in a Pharos PX (Bio-Rad, Hercules, CA, USA). One unit of polymerase activity was defined as the amount of enzyme that incorporated 1 nmol of dTTP into an acid-insoluble material in 10 min at 37 °C.

### 2.6. Droplet Digital PCR

The concentrations of DNA and RNA in the obtained standards were refined by a digital PCR using the QX200™ Droplet Digital™ PCR System (Bio-Rad, Hercules, CA, USA) according to the manufacturer’s instructions. The reactions were carried out in a total volume of 20 µL containing the DNA under examination (approximately 10^3^ copies per 20 µL), 1x ddPCR master-mix (Bio-Rad, Hercules, CA, USA), 300 nM oligonucleotide primers and probes: E_Sarb_F/R/P for SARS-CoV-2 [44], and MS2-5_F/R/P for MS2 phage (Table 2). For droplet generation, 20 µL of the PCR mix and 70 µL of the droplet generation oil were placed into corresponding wells of the DG8 cartridge, and the droplets were obtained in a droplet generator. Then, 40 µL of the obtained droplets were transferred to the 96-well PCR plate, foil-sealed, and placed into the thermocycler. The amplification was performed using the following program: 96 °C for 10 min, followed by 45 cycles of 96 °C for 30 s, 58 °C for 60 s, with final heating for 10 min at 98 °C. The ramp rate was 2 °C/s for all of the steps. The droplets were analyzed by the droplet reader, and the data obtained were processed by the QuantaSoft package (Bio-Rad, Hercules, CA, USA).

### 2.7. Real-Time Reverse-Transcription Loop-Mediated Isothermal Amplification (RT-LAMP)

The reaction mixture for LAMP (20 µL) contained 1× reaction buffer for Bst-polymerase (20 mM Tris-HCl pH 8.8, 10 mM (NH_4_)_2_SO_4_, 50 mM KCl, 0.1% Tween-20, 8 mM MgSO_4_), 1.25 mM each dNTP, 0.4 µM each external primer (F3/B3), 0.8 µM loop primers (LF/BF), 1.6 µM internal primers (FIP/BIP) (Table 2), DNA or RNA template (the type and amount of the template are given below), two units of Gss-polymerase from *Geobacillus* sp. 777 [46], and 1 µM intercalating dye SYTO-82. The amount of reverse transcriptases varied in the range of 0.4–20 U/reaction. For the exact amount of RTs for each experiment, one can refer to the Results section. Each experiment was conducted in three independent replicates, and each run included a no-template control and a no reverse transcriptase control. RT-LAMP was performed in the CFX96 thermocycler (Bio-Rad, Hercules, CA, USA) using a two-step program with the initial reverse transcription, followed by LAMP in real-time mode. The exact time and temperature of the reverse transcription step were varied and are specified below in the Results section for each experiment. The LAMP program included the following steps: 90 cycles of primer annealing and elongation, each at 62 °C for 20 s with the registration of the fluorescence signal in the HEX channel and post-amplification melting of the amplification products in the range of 70–95 °C. Tt values (time-to-threshold, time interval before the intersection between an amplification curve and a threshold line) were calculated after each run and were used to assess the RT-LAMP efficacy.

## 3. Results

In the present work, the enzymes to be studied were selected according to their popularity and improvements by various approaches, including different mutations and fusion with DNA-binding proteins. We compared in RT-LAMP several M-MuLV RT variants: two of the most popular commercial enzymes, Superscript III and Superscript IV; RNAse H- M-MuLV RT (analog of Superscript II); RT mut (M-MuLV RT with previously described L139P, D200N, T330P mutations), fusions of RNAse H-M-MuLV RT and RT mut with DNA-binding protein Sto7d from *Sulfolobus tokodaii*. The features of the enzymes are listed in Table 1.

To evaluate the performance of various RTs, we used a previously designed LAMP assay for SARS-CoV-2 RNA detection [46]. Briefly, two primer sets for conservative regions of SARS-CoV-2 genomic RNA and MS2 phage genomic RNA were chosen and used for RT-LAMP in a real-time mode (Table 2). As targets for the LAMP, we selected a conserved region of SARS-CoV-2 genomic RNA encoding the N protein (GenBank ID NC_045512.2) and a region of MS2 phage genomic RNA (GenBank ID NC_001417). The primers were designed according to the recommendations on the primerexplorer.jp website. We prepared a plasmid control for RT-LAMP based on pBlueScript II SK (+) vector and a 200 bp SARS-CoV-2 genome fragment. The RNA standards were synthesized using the in vitro transcription of the SARS-CoV-2 plasmid standard or were prepared from MS2 genomic RNA. All of the controls were quantified by digital PCR on the QX200 platform. 

### 3.1. Titration of RTs in RT-LAMP

**Figure 1:**
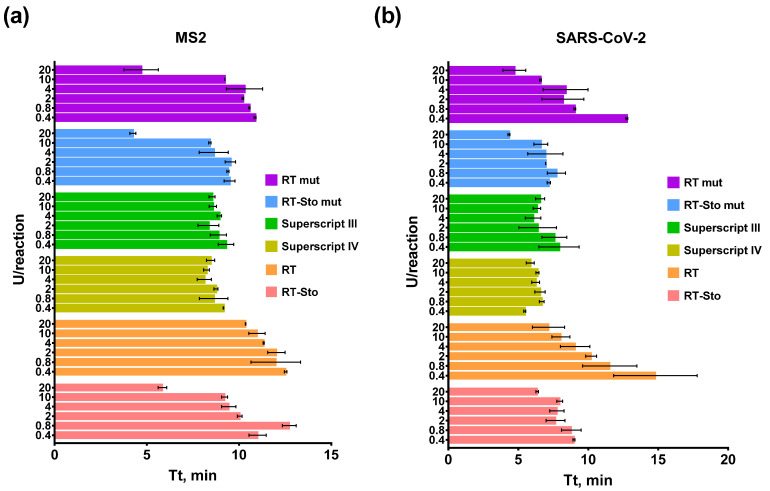
RT-LAMP with various amounts of RTs and primers for MS2 (**a**) and SARS-CoV-2 (**b**). The temperature of the reverse transcription step was 42 °C. Each enzyme is marked by the color specified in the legend. SARS-CoV-2 RNA fragment and MS2 genomic RNA were added in reactions to a final concentration of 10^3^ copies per reaction. The time-to-threshold (Tt) values are presented on the X-axis, and the number of enzyme units per reaction is on the Y-axis. Each experiment was triplicated, with error bars representing one SD.

Initially, we titrated RTs in RT-LAMP to assess the range of RT concentrations that would provide a sufficient RT-LAMP efficacy. This parameter could be crucial as excess enzymes might lead to a spurious non-specific amplification (false-positive results) and enzyme shortage might cause a slow and inefficient reaction (false-negative results). All of the enzymes were titrated at a range of 0.4–20 U/reaction. The template concentration for RT-LAMP, SARS-CoV-2 RNA fragment, and MS2 genomic RNA was 10^3^ copies per reaction. Each experiment was conducted in three independent replicates, with each replicate including a no-template control and a no-reverse transcriptase control. The results of the enzyme titration are presented in Figure 1 and Supplementary Figure S1.

**Figure 2:**
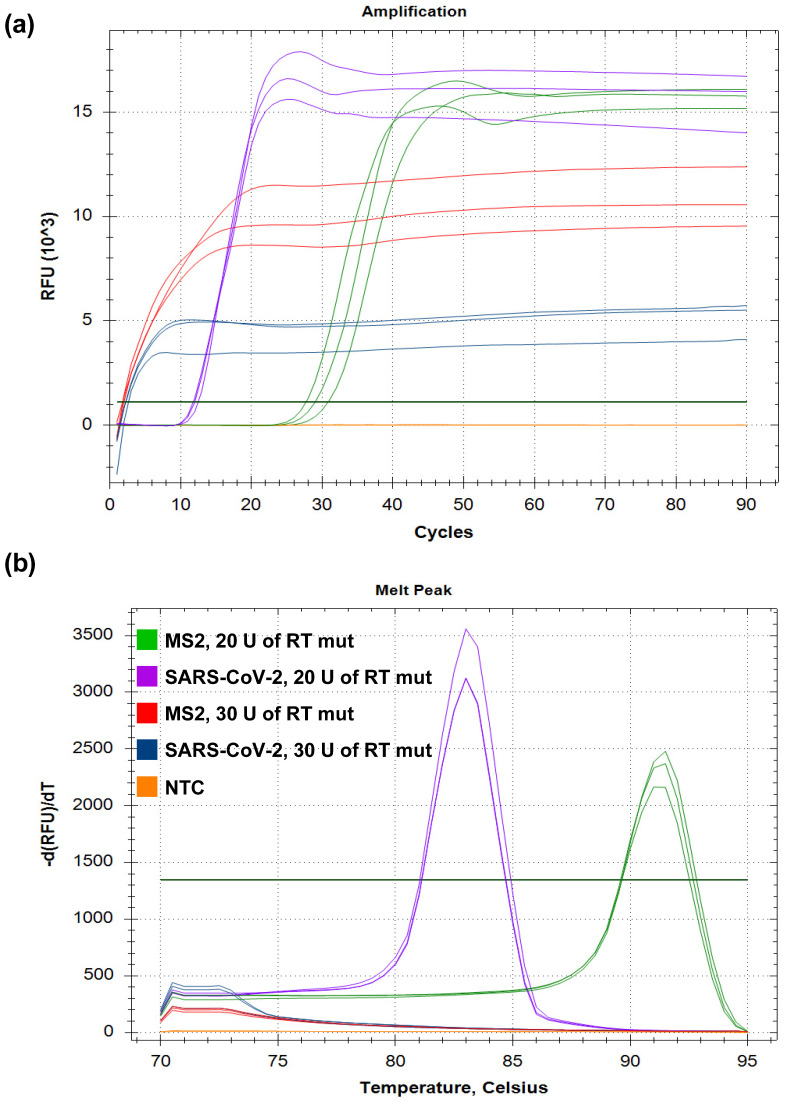
RT-LAMP plot with amplification curves (**a**) and melting analysis (**b**). The temperature of the reverse transcription step was 42 °C. SARS-CoV-2 RNA fragment and MS2 genomic RNA were added in reactions to a final concentration of 10^3^ copies per reaction. RT-LAMP for MS2 with 20 U of RT mut is marked in green, and RT-LAMP for SARS-CoV-2 with 20 U of RT mut is designated in violet. RT-LAMP for MS2 with 30 U of RT mut is marked in red, RT-LAMP for SARS-CoV-2 RT-LAMP for MS2 with 30 U of RT mut is designated in blue, and NTC is marked in orange.

All of the RTs performed the reverse transcription step of RT-LAMP when added in the reaction in a similar range of 0.4–20 U/reaction. However, the efficacy of RTs was different. Thus, the reaction rate of the native M-MuLV RT was lower than that of the other enzymes in both model RT-LAMP assays, as manifested by the higher Tt values. The efficacy of RT-LAMP with Superscript III and Superscript IV was the same in the whole range of 0.4–20 U/reaction. Non-specific amplification products appeared in RT-LAMP for MS2 and SARS-CoV-2 with homemade enzymes at a concentration of more than 20 U per reaction, with the single exception being RT (Supplementary Figure S1). In other cases, no non-specific melting peaks appeared after the amplification (Figure 2). To avoid contamination by LAMP products, gel electrophoresis was not used in further experiments, as LAMP products are produced in enormous amounts and are persistent in laboratory equipment. In addition, the end-point analysis of the saturated reaction mixes after amplification does not provide information about the actual kinetic of LAMP.

Thus, commercial enzymes were suitable for RT-LAMP in a range of 0.4–20 U/reaction, while the homemade enzymes were 0.4–20 U/reaction. In the latter experiments, the final concentration of all of the enzymes was 10 U/reaction.

### 3.2. Optimal Temperature of the Reverse Transcription Step in RT-LAMP

**Figure 3:**
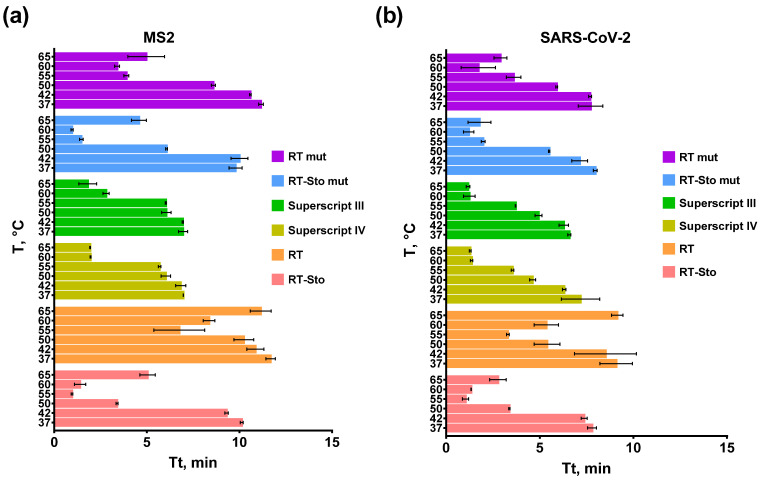
RT-LAMP at different reverse transcription temperatures and primers for MS2 (**a**) and SARS-CoV-2 (**b**). Each enzyme is marked by the color specified in the legend. SARS-CoV-2 RNA fragment and MS2 genomic RNA were added in the reactions to a final concentration of 10^3^ copies per reaction. Time-to-threshold (Tt) values are presented on the X-axis, and the temperature of the reserve transcription is shown on the Y-axis. Each experiment was triplicated, with error bars representing one SD.

In the next step of comparison, we defined the optimal temperature for the reverse transcription step in the RT-LAMP for each enzyme. Various RTs have different temperature optimums, depending on the exact alteration composition. A suboptimal reaction temperature could result in a lower reaction speed as the enzyme might lose optimal conformation or be thermally denatured. The template secondary structure at a low reaction temperature could stall the enzyme progression. The temperature of reverse transcription in RT-LAMP varied in the range of 37–65 °C. The template concentration for RT-LAMP, SARS-CoV-2 RNA fragment, and MS2 genomic RNA was 10^3^ copies per reaction; the final concentration of all of the enzymes was 10 U/reaction. Each experiment was conducted in three independent replicates, with each replicate including a no-template control and a no-reverse transcriptase control. The results are presented in Figure 3.

Commercial enzymes, Superscript III and Superscript IV, were the most active at the temperature range of 60–65 °C. These enzymes could work at a higher temperature. The annealing temperature of the LAMP primers used was a limiting factor preventing testing at higher temperatures because all of the LAMP primers were designed to have T_annealing_ at a range of 60–62 °C. The optimal temperature was 60 °C for RT mut and RT-Sto mut, and 55 °C for RT and RT-Sto. As shown before, the optimal temperature for chimeric enzymes was similar to the cognate RTs, despite the presence of Sto7d. Thus, commercial enzymes enriched with mutations demonstrated a higher thermostability than the chimeric enzymes or enzymes with L139P, D200N, and T330P mutations. In further experiments, the reverse transcription temperature was 55 °C for RT and RT-Sto and 60 °C for all of the other enzymes.

### 3.3. Optimal Time of the Reverse Transcription Step in RT-LAMP

**Figure 4:**
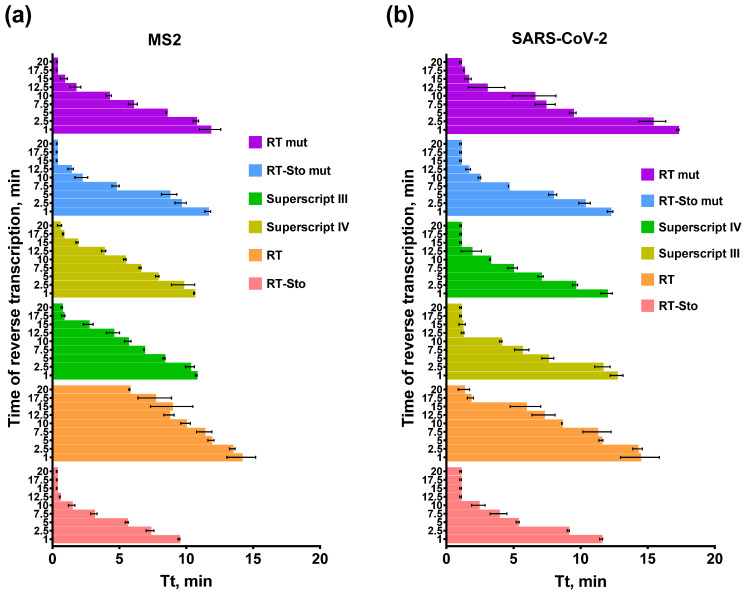
RT-LAMP with different times for the reverse transcription step and primers for MS2 (**a**) and SARS-CoV-2 (**b**). The temperature of the reverse transcription step was 55 °C for RT and RT-Sto and 60 °C for the remaining enzymes. Each enzyme is marked by the color specified in the legend. SARS-CoV-2 RNA fragment and MS2 genomic RNA were added in the reactions to a final concentration of 10^3^ copies per reaction. Time-to-threshold (Tt) values are presented on the X-axis, and the time of the reserve transcription step is shown on the X-axis. Each experiment was triplicated, with error bars representing one SD.

The optimal time for reverse transcription could be crucial for diagnostic tests because they often require a high throughput capacity for mass-scale testing. Thus, machine time could be a limiting factor, particularly in pandemic situations or testing in crowded places. Normally, an excessive number of RTs are used to catalyze reverse transcription in RT-PCR or RT-LAMP. However, an excess of enzymes can lead to false-positive test results. In the samples with a low abundance of target RNA, an insufficient reverse transcription time could result in false-negative test results. The fast enzyme could speed up the analysis and increase the throughput capacity of the diagnostic laboratories. To evaluate the optimal time of the reverse transcription, we changed this parameter from 1 to 20 min. The template concentration for RT-LAMP, SARS-CoV-2 RNA fragment, and MS2 genomic RNA was 10^3^ copies per reaction. Each experiment was conducted in three independent replicates, with each replicate including a no-template control and a no-reverse transcriptase control. The results of the experiments are presented in Figure 4.

As expected, increasing the reverse transcription time led to a lower Tt and faster LAMP results. Thus, prolonged reverse transcription decreased the Tt values for 5–10 min, depending on the enzyme. Further prolongation of the reverse transcription reduced Tt values to less than 1 min. However, in that case, the overall time of RT-LAMP remained the same. Tt values of less than 1 min were caused by Gss-polymerase activity during the reverse transcription step. Thus, the LAMP amplicons synthesized during the reverse transcription step created a detectable fluorescent signal at the beginning of the LAMP step. RT-Sto and RT-Sto mut demonstrated a higher rate of reverse transcription with RT-LAMP for MS2 but not with RT-LAMP for SARS-CoV-2. An additional DNA-binding domain facilitatedthe enzyme performance on the template with a complex secondary structure. In most cases, RT-LAMP with RT showed the highest Tt, indicating the lowest reaction speed with this enzyme. For further experiments, we chose 10 min for reverse transcription as a reasonable compromise for reducing the overall amplification time, including both reverse transcription and LAMP steps.

### 3.4. RNA Titration in RT-LAMP

**Figure 5:**
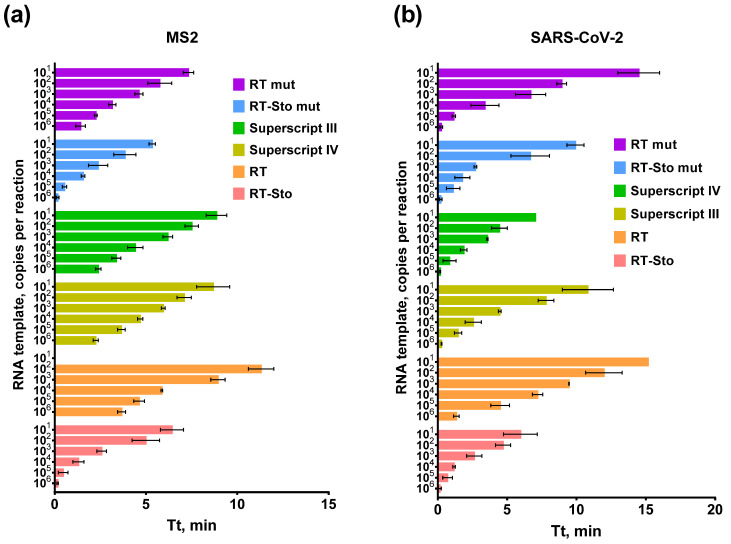
RT-LAMP with RNA template titration and primers for MS2 (**a**) and SARS-CoV-2 (**b**). The temperature of the reverse transcription step was 55 °C for RT and RT-Sto and 60 °C for the remaining enzymes. Each enzyme is marked by the color specified in the legend. Time-to-threshold (Tt) values are presented on the X-axis and the RNA template concentration is shown on the Y-axis. Each experiment was triplicated, with error bars representing one SD.

Analytical sensitivity, defined as the lowest detectable analyte concentration, is one of the crucial parameters for diagnostics tests. Insufficient sensitivity could result in false-negative results. Thus, in the case of viral testing, patients would not receive the appropriate therapy and would continue to spread the virus. To evaluate the ability of RTs to use various amounts of RNA as templates, we titrated the SARS-CoV-2 RNA fragment and MS2 genomic RNA in the range of 10^6^–10^1^ copies per reaction. Each experiment was conducted in three independent replicates, with each replicate having a no-template control and a no-reverse transcriptase control. The results of titration are presented in Figure 5.

Most of the RTs demonstrated a similar efficacy for various concentrations of a template. Six orders of magnitude of RNA concentration generated a positive amplification signal. Notably, the efficiency of RT-LAMP with SARS-CoV-2 was higher than with MS2. The latter could be caused by the complex secondary structure of the MS2 genomic RNA, as mentioned above for the experiment with the time for the reverse transcription. Among all of the enzymes, only RT performed RT-LAMP slower, similarly to previous experiments.

### 3.5. Inhibitors in RT-LAMP

**Figure 6:**
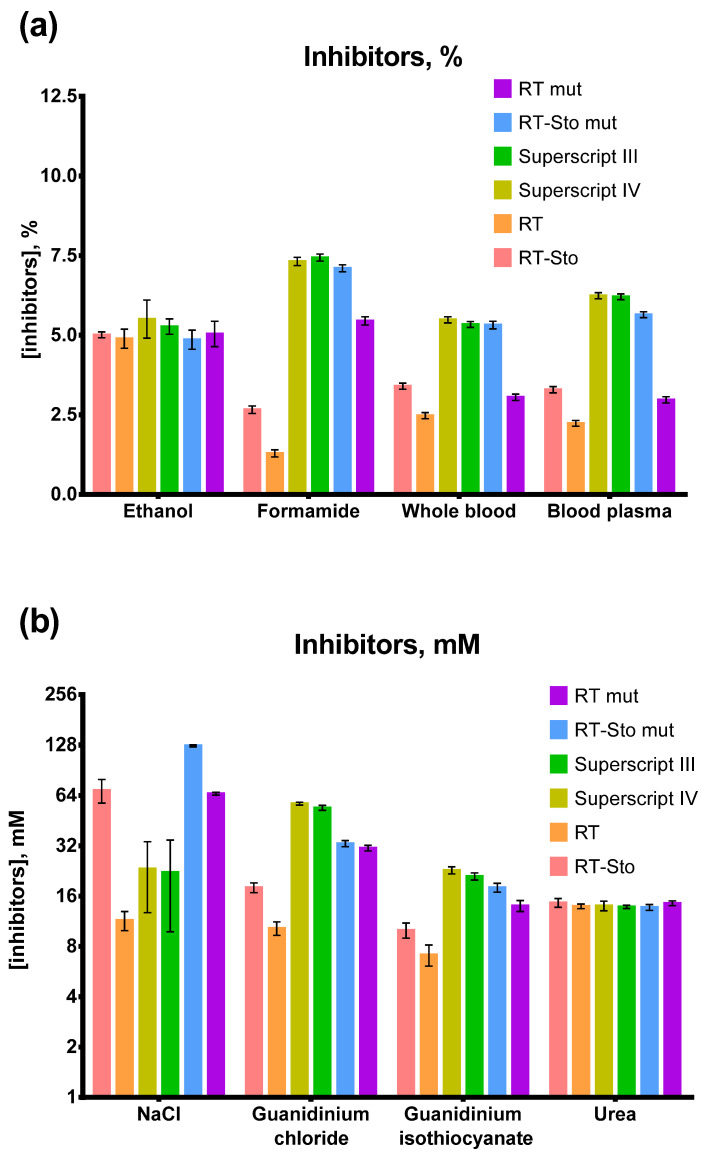
RT-LAMP with various inhibitors. Substances tested as inhibitors in RT-LAMP: (**a**) ethanol, formamide, human whole blood, and human blood plasma; (**b**) NaCl, guanidinium isothiocyanate, guanidinium chloride, and urea. The temperature of the reverse transcription step was 55 °C for RT and RT-Sto and 60 °C for the remaining enzymes. SARS-CoV-2 RNA fragment and MS2 genomic RNA were added in the reactions to a final concentration of 10^3^ copies per reaction. Each enzyme is marked by the color specified in the legend. IC_50_ values for different inhibitors were calculated and plotted on the Y-axis. Each experiment was triplicated, with error bars representing one SD.

Various substances originating from samples or RNA purification procedures could inhibit the catalysis of RTs. Human whole blood and blood plasma, urea, humic acids, ethanol, NaCl, and guanidinium are among these inhibitors. In most cases, laboratory purification protocols allow DNA/RNA samples to be obtained free from traces of inhibitors. However, such methods could be unavailable in the case of point-of-care testing, which is the domain of LAMP, and other approaches for isothermal amplification. To assess the ability of different RTs to perform RT-LAMP in the presence of inhibitors, we titrated several inhibiting substances in RT-LAMP reactions: human whole blood, blood plasma, urea, ethanol, NaCl, salts of guanidinium, formamide, and NaCl. The template concentration for RT-LAMP, SARS-CoV-2 RNA fragment, and MS2 genomic RNA was 10^3^ copies per reaction. Each experiment was conducted in three independent replicates, with each replicate including a no-template control and a no reverse transcriptase control. The calculated IC 50 values for each inhibitor are presented in Figure 6.

As anticipated, the presence of mutations and an additional DNA binding domain increased tolerance to inhibitors of the respective altered RTs. However, the scale of the positive effect varied depending on the inhibitor. Thus, Sto7d or triple mutations allowed M-MuLV to work at a six-fold higher concentration of NaCl. However, M-MuLV RT alterations broadened the working concentration range by only 1.5–3-fold for whole blood, blood plasma, and guanidinium. All of the enzymes were equally sensitive to the presence of ethanol and urea. Thus, mutations and Sto7d mitigate the effect of inhibitors related to enzyme-template binding while being ineffective against other substances. Highly mutated enzymes were superior to most of the other inhibitors. The single exception was NaCl, when RT mut and RT-Sto mut outperformed commercial RTs. The inhibitory effect of guanidinium isothiocyanate was higher than that of guanidinium chloride.

## 4. Discussion

Reverse transcriptases allowing RNA to be analyzed without the constant risk of degradation are one of the cornerstones of modern biology. With numerous RTs being characterized and marketed [5,6,7,8], M-MuLV RT and its altered cognates remain the most widely used. The properties of mutant M-MuLV RTs and the efficacy of various RTs in practical applications, including PCR and NGS, have been reported in multiple articles [9,10,11,12,13,14,15,16,17]. A direct comparison of various RTs under similar conditions allows the optimal enzyme to be selected without the pitfalls of different modeling approaches used in different laboratories. However, the performance of RTs in some widely used methods still needs to be investigated. Thus, the demand for point-of-care tests in the post-COVID-19 era entailed a growing interest in various isothermal approaches for nucleic acid amplification [25]. One of the most popular techniques is LAMP, and dozens of LAMP-based tests have been devised for testing different pathogens [25,31,32,33,34,35,36,47]. However, the efficacy of various RTs to perform RT-LAMP remains unknown.

In the present work, we focused on comparing popular highly mutated commercial M-MuLV variants and homemade enzymes harboring only three mutations or an additional DNA-binding domain. The intention was to determine the effect of multiple alterations in M-MuLV RT on its actual performance in RT-LAMP. Currently, most vendors provide altered M-MuLV RT with several patented mutations. The number of mutations increases over time, and the latest versions of M-MuLV can harbor up to dozens of altered amino acids. Thus, Superscript II (1988) was a truncated M-MuLV RT without RNAse H domain [48], Superscript III (2006) contained seven mutations [49], and Superscript IV (2014) had 16 mutations [50]. Most of these mutations are in sites interacting with a template or inactivating RNAse H domain, increasing the thermal stability and temperature optimum [17,22,51,52]. The effect of several mutations on RT performance is believed to be additive as amino acid substitutions are independent of each other. However, it remains unknown whether a large number of mutations are necessary for the desirable efficacy of reverse transcription in practical applications such as RT-LAMP.

Here, we made a preliminary comparison of RTs with various amounts of mutations or with an additional DNA-binding domain in RT-LAMP. As a control, we chose the truncated M-MuLV RT without the RNAse H domain, which is essentially the first version of Superscript II. RT mut, M-MuLV RT with three mutations (L139P, D200N, and T330P) claimed by Fermentas in 2008 [17,53], served as the enzyme with a low number of alterations. Superscript III and Superscript IV were added to the panel of studied RTs as highly mutated RTs, which are popular on the market. Additionally, chimeric M-MuLV RT and RT mut with fused Sto7d were studied. Thus, specimens of RTs with various mutations and an additional domain were compared in a single study with uniform methods.

All of the enzymes were synthesized cDNA in concentrations of 0.4–20 U/reaction. However, 0.4 U/reaction of RT and RT mut worked visibly slower, and at more than 20 U/reaction, non-specific products accumulated during the reverse transcription step. An insufficient amount of RT leads to slower amplification and potential false-negative results, especially when the concentration of the target RNA is low [54,55]. In turn, RT excess could result in spurious amplification and false-positive testing [56], an example being the appearance of non-specific amplification products mentioned above. These observations indicate the need for careful enzyme titration to prevent both types of false results. Another explanation of false-positive results is non-specific amplification during LAMP caused by the presence of six primers in a high concentration. However, no such issues were noted in the present work. It is likely that in many cases, false-positives were the result of contamination by amplicons from previous runs. This artificial DNA is found on virtually all laboratory surfaces and equipment, with its impact on testing often underestimated [39]. Gel electrophoresis, sequencing of amplification products, and any other operations related to opening reaction tubes after LAMP could lead to persistent contamination [57]. The risk of amplicon spreading is higher when these procedures are performed in the same room and with the same equipment as for LAMP. Thus, proper handling of all of the reagents and cautious regular control of the reagent purity could have prevented and would prevent the problems related to the sudden appearance of positive LAMP results.

As expected, the optimal temperature for RTs was the same as previously reported. A slightly higher optimal temperature, 55 °C, was observed only for RT and RT-Sto. RNAse H-deficient M-MuLV RT was reported to have an optimum temperature of 42–50 °C [19,58]. The presence of Sto7d does not affect the thermal stability and optimal temperature of chimeric enzymes [20]. A plausible explanation for this apparent higher thermal stability of non-mutated RTs could be that M-MuLV RT can work at 50–55 °C for a short time, enough to produce enough of the template for LAMP. Higher reaction temperature leads to denaturation of RNA template secondary structure, facilitating cDNA synthesis [11]. Thus, despite thermal inactivation, thermolabile enzymes can synthesize cDNA using denatured templates.

RTs with three mutations demonstrated the same thermal optimum, 60 °C, and were less thermostable than highly mutated superscripts. Thus, multiple mutations were proven to increase the thermal stability of M-MuLV RT in practical applications. The optimum temperature for Bst-like polymerases was in the range of 60–65 °C. Therefore, a working temperature of 60 °C was sufficient for RTs working with Bst-polymerase in a single enzymatic reaction.

The overall time for analysis could be a critical parameter in a situation when many samples should be tested in a limited time. In this sense, rapid amplification attained with fast enzymes is beneficial and saves valuable machine time. Among all of the studied enzymes, M-MuLV RT H- was the slowest, while its chimeric cognate with Sto7d demonstrated a speed at the level of commercial RTs. The difference in reaction speed between other enzymes was much lower, regardless of the mutation number and presence of Sto7d. Fused enzymes demonstrated a higher reaction rate only with RT-LAMP for MS2. These observations could be a result of the intact catalytic rate and enhanced affinity to a template. Most reported mutations in M-MuLV RT affect its interaction with a template, but not the catalytic properties [16,17,18]. The additional DNA-binding domain with a flexible linker provides a second point of binding with a template, preventing complete dissociation of the enzyme–template complex. Thus, chimeric enzymes could be superior to their mutated counterparts when the template is readily able to form a complex secondary structure.

All chimeric and mutant enzymes showed a similar ability to produce cDNA using a minuscule quantity of RNA template. Only M-MuLV RT H- was unable to convert a low concentration of MS2 phage genomic RNA. This finding agrees with the previous observation of the reaction rate, as the enhanced affinity of altered M-MuLV RTs allowed them to utilize smaller quantities of a complex template. Interestingly, highly mutated enzymes were not superior to the triple mutant or chimeric RTs. Presumably, a slight increase in affinity to a template was enough to increase the overall sensitivity of reverse transcription. Further alterations to RTs had no positive effect on the ability to work with low template concentrations.

All RTs demonstrated similar values or a minor difference in optimal concentration, reaction rate, and temperature, as well as the ability to utilize a small amount of template. However, highly mutated RTs were superior in their tolerance to various inhibitors. One possible explanation for this could be the higher affinity to a template masked in other experiments. Only when enzymes were tested close to their limits did the stronger binding allow for mitigating the effect of the inhibitor substances. The exception was NaCl, which acts as a screen for charged surfaces and prevents the interaction of proteins and nucleic acids [59,60]. Some mutations in Upper Index III and Upper Index IV appeared to have made these enzymes sensitive to NaCl, but the exact mechanism of such specific inhibition remains unknown.

Several limitations of the present study need to be mentioned. The available group of commercial RTs is broad and cannot be covered in a single study. Other RTs, such as WarmStart and Maxima RT, could demonstrate a different efficacy in RT-LAMP, depending on the actual composition of mutations. Here, we only presented a short glimpse of the problem, and further extensive studies are needed to evaluate the performance of numerous RTs in practical applications. Another matter of concern is the choice of model systems. The efficacy of different enzymes could vary depending on the GC content, secondary structures, and primer concentrations [61,62,63]. However, a more complex problem is the reaction buffer formulation. The ionic strength, presence, and concentration of various ions and other compounds, such as DMSO and betaine, define the rate of enzymatic catalysis [64,65]. A suboptimal buffer composition could bias the results of the present work. Conversely, RT-LAMP assumes the reaction buffer to be used for Bst-polymerase, suggesting that RTs need to be able to work in the prescribed buffer. The composition of the reaction buffer remains a separate matter of concern that needs to be considered when developing diagnostic tests. 

To sum up, a set of mutant M-MuLV RTs was compared in RT-LAMP, defining the enzyme efficiency in two model systems. Several parameters were evaluated: optimal temperature, enzyme concentration, time for reverse transcription, ability to utilize small amounts of RNA templates, and tolerance to inhibitors. As expected, mutated RTs demonstrated a higher efficacy than intact M-MuLV RT.

## 5. Conclusions

To the best of our knowledge, we report here on the first direct comparison in RT-LAMP of various reverse transcriptases originating from M-MuLV RT. The enzymes studied included highly mutated commercial RTs, triple mutant M-MuLV RT, fusion RTs with Sto7d, and H-M-MuLV RT. Several parameters were assessed, such as the optimal temperature, enzyme concentration, time of reverse transcription, ability to utilize small amounts of RNA templates, and tolerance to inhibitors. Unlike fused Sto7d, mutations were proven to increase the optimal temperature of the reverse transcription step in RT-LAMP. For either Sto7d, three or more mutations had a similar positive effect on the efficacy of RTs, decreasing the time for reverse transcription and enhancing the ability to utilize low concentrations of RNA templates. Highly mutated commercial enzymes proved to be superior to triple mutants and chimeric RTs in terms of tolerance to human whole blood, blood plasma, and guanidinium. However, they were found to be more sensitive to high concentrations of NaCl. The comparison of various RTs presented here is expected to be helpful for selecting the optimal enzyme to develop novel diagnostic tests based on LAMP.

## Figures and Tables

**Table 1:** Reverse transcriptases and their features.

Name	Alteration
RT	Truncated RNAse H domain
RT-Sto	Truncated RNAse H domain, fusion with Sto7d protein
RT mut	Truncated RNAse H domain, mutations L139P, D200N, T330P
RT-Sto-mut	Truncated RNAse H domain, mutations L139P, D200N, T330P, fusion with Sto7d protein
Superscript III	Mutations H204R, T306K, F309N, V223H, D524G, E562Q, and D583N
Superscript IV	Mutations P51L, S67R, E69K, T197A, H204R, E302K, F309N, W313F, T330P, L435G, N454K, D524G, D583N, H594Q, D653N, and L671P

**Table 2:** List of oligonucleotide primers and probes [45].

Name	5′-Sequence-3′
CoR2-F2	TGCAACTGAGGGAGCCTTG
CoR2-B2	TGGAGTTGAATTTCTTGAACTG
CoR2-LF	CGGCAGTCAAGCCTCTTCTC
CoR2-LB	ATTGTTAGCAGGATTGCGGGT
CoR2-FIP	GGAAGTTGTAGCACGATTGCAGATACACCAAAAGATCACATTGG
CoR2-BIP	GCTTCTACGCAGAAGGGAGCATGCGACTACGTGATGAGGAA
MS2-2-F3	TGCCTGTAAGGAGCCTGAT
MS2-2-B3	TGAGCGGATACGATCGAGAT
MS2-2-LB	GTCTATACCAACGGATTTGAGCC
MS2-2-LF	GCATCCGATTCCATCTCCGAT
MS2-1-FIP	CTCCTGAGGGAATGTGGGAACCCCGGCGTGCGCGTTAT
MS2-1-BIP	GCCAGCGAGCTCTCCTCGGGCACCCGTGCTCTTTCGA
E_Sarb_F	ACAGGTACGTTAATAGTTAATAGCGT
E_Sarb_R	ATATTGCAGCAGTACGCACACA
E_Sarb_P	HEX-ACACTAGCCATCCTTACTGCGCTTCG-BHQ2
MS2-5-F	GTACGAGGAGAAAGCCGGTTTC
MS2-5-R	GTTCTGCGGCACTTCGATG
MS2-5-P	FAM-TCCCTCGACGCACGCTCCTGCT-BHQ1

## Data Availability

The data presented in this study are available upon request from the corresponding author.

## Supplementary Materials

The following supporting information can be downloaded at: https://www.mdpi.com/article/10.3390/biology11121809/s1. Figure S1: RT-LAMP with various amounts of RTs and primers for MS2.
