# Supplementary material for: Comparison of Reverse Transcriptase (RT) Activities of Various M-MuLV RTs for RT-LAMP Assays
Source: Biology (Basel). 2022 Dec 13;11(12):1809. doi: 10.3390/biology11121809 (PMC9775983; doi:10.3390/biology11121809)
Supplement: Supplementary file 1 [file biology-11-01809-s001.zip › biology-2079741-supplementary.pdf]

Sequences and maps of cloned reverse transcriptases

pBlueScript-CoV-2

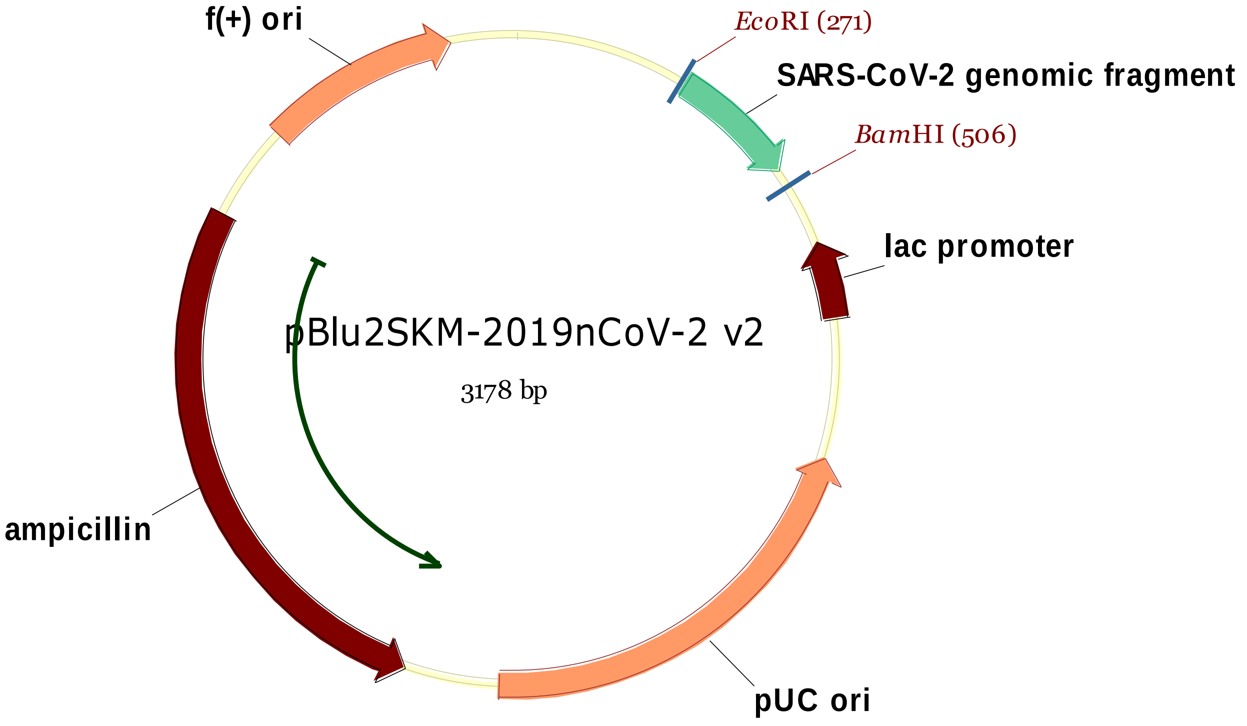

tttaacaaaatattaacgcttacaatttccattcgccattcaggctgcgcaactgttgggaagggcgatcggtgcgggcctcttcgctattacgccagctggc
gaaagggggatgtgctgcaaggcgattaagttgggtaacgccagggttttcccagtcacgacgttgtaaaacgacggccagtgagcgcgcgtaatacgact
cactatagggcgaattgggtaccgggccccccctcgaggtcgacggtatcgataagcttgatatcgaattcttgcaactgagggagccttgaatacaccaaa
agatcacattggcacccgcaatcctgctaacaatgctgcaatcgtgctacaacttcctcaaggaacaacattgccaaaaggcttctacgcagaagggagca
gaggcggcagtcaagcctcttctcgttcctcatcacgtagtcgcaacagttcaagaaattcaactccaaagcttctgcagacgcgtcgacgtcatatggatcc
actagttctagagcggccgccaccgcggtggagctccagcttttgttccctttagtgagggttaattgcgcgcttggcgtaatcatggtcatagctgtttcctgt
gtgaaattgttatccgctcacaattccacacaacatacgagccggaagcataaagtgtaaagcctggggtgcctaatgagtgagctaactcacattaattgc
gttgcgctcactgcccgctttccagtcgggaaacctgtcgtgccagctgcattaatgaatcggccaacgcgcggggagaggcggtttgcgtattgggcgctct
tccgcttcctcgctcactgactcgctgcgctcggtcgttcggctgcggcgagcggtatcagctcactcaaaggcggtaatacggttatccacagaatcagggg
ataacgcaggaaagaacatgtgagcaaaaggccagcaaaaggccaggaaccgtaaaaaggccgcgttgctggcgtttttccataggctccgcccccctga
cgagcatcacaaaaatcgacgctcaagtcagaggtggcgaaacccgacaggactataaagataccaggcgtttccccctggaagctccctcgtgcgctctc
ctgttccgaccctgccgcttaccggatacctgtccgcctttctcccttcgggaagcgtggcgctttctcatagctcacgctgtaggtatctcagttcggtgtaggt
cgttcgctccaagctgggctgtgtgcacgaaccccccgttcagcccgaccgctgcgccttatccggtaactatcgtcttgagtccaacccggtaagacacgac
ttatcgccactggcagcagccactggtaacaggattagcagagcgaggtatgtaggcggtgctacagagttcttgaagtggtggcctaactacggctacact
agaaggacagtatttggtatctgcgctctgctgaagccagttaccttcggaaaaagagttggtagctcttgatccggcaaacaaaccaccgctggtagcggt
ggtttttttgtttgcaagcagcagattacgcgcagaaaaaaaggatctcaagaagatcctttgatcttttctacggggtctgacgctcagtggaacgaaaact
cacgttaagggattttggtcatgagattatcaaaaaggatcttcacctagatccttttaaattaaaaatgaagttttaaatcaatctaaagtatatatgagtaa
acttggtctgacagttaccaatgcttaatcagtgaggcacctatctcagcgatctgtctatttcgttcatccatagttgcctgactccccgtcgtgtagataact
acgatacgggagggcttaccatctggccccagtgctgcaatgataccgcgagacccacgctcaccggctccagatttatcagcaataaaccagccagccgg
aagggccgagcgcagaagtggtcctgcaactttatccgcctccatccagtctattaattgttgccgggaagctagagtaagtagttcgccagttaatagtttg
cgcaacgttgttgccattgctacaggcatcgtggtgtcacgctcgtcgtttggtatggcttcattcagctccggttcccaacgatcaaggcgagttacatgatc
ccccatgttgtgcaaaaaagcggttagctccttcggtcctccgatcgttgtcagaagtaagttggccgcagtgttatcactcatggttatggcagcactgcata
attctcttactgtcatgccatccgtaagatgcttttctgtgactggtgagtactcaaccaagtcattctgagaatagtgtatgcggcgaccgagttgctcttgcc
cggcgtcaatacgggataataccgcgccacatagcagaactttaaaagtgctcatcattggaaaacgttcttcggggcgaaaactctcaaggatcttaccgc

tgttgagatccagttcgatgtaacccactcgtgcacccaactgatcttcagcatcttttactttcaccagcgtttctgggtgagcaaaaacaggaaggcaaaa
tgccgcaaaaaagggaataagggcgacacggaaatgttgaatactcatactcttcctttttcaatattattgaagcatttatcagggttattgtctcatgagcg
gatacatatttgaatgtatttagaaaaataaacaaataggggttccgcgcacatttccccgaaaagtgccacctgacgcgccctgtagcggcgcattaagcg
cggcgggtgtggtggttacgcgcagcgtgaccgctacacttgccagcgccctagcgcccgctcctttcgctttcttcccttcctttctcgccacgttcgccggct
ttccccgtcaagctctaaatcgggggctccctttagggttccgatttagtgctttacggcacctcgaccccaaaaaacttgattagggtgatggttcacgtagt
gggccatcgccctgatagacggtttttcgccctttgacgttggagtccacgttctttaatagtggactcttgttccaaactggaacaacactcaaccctatctcg
gtctattcttttgatttataagggattttgccgatttcggcctattggttaaaaaatgagctgatttaacaaaaatttaacgcgaat

pBlueScript-MS2

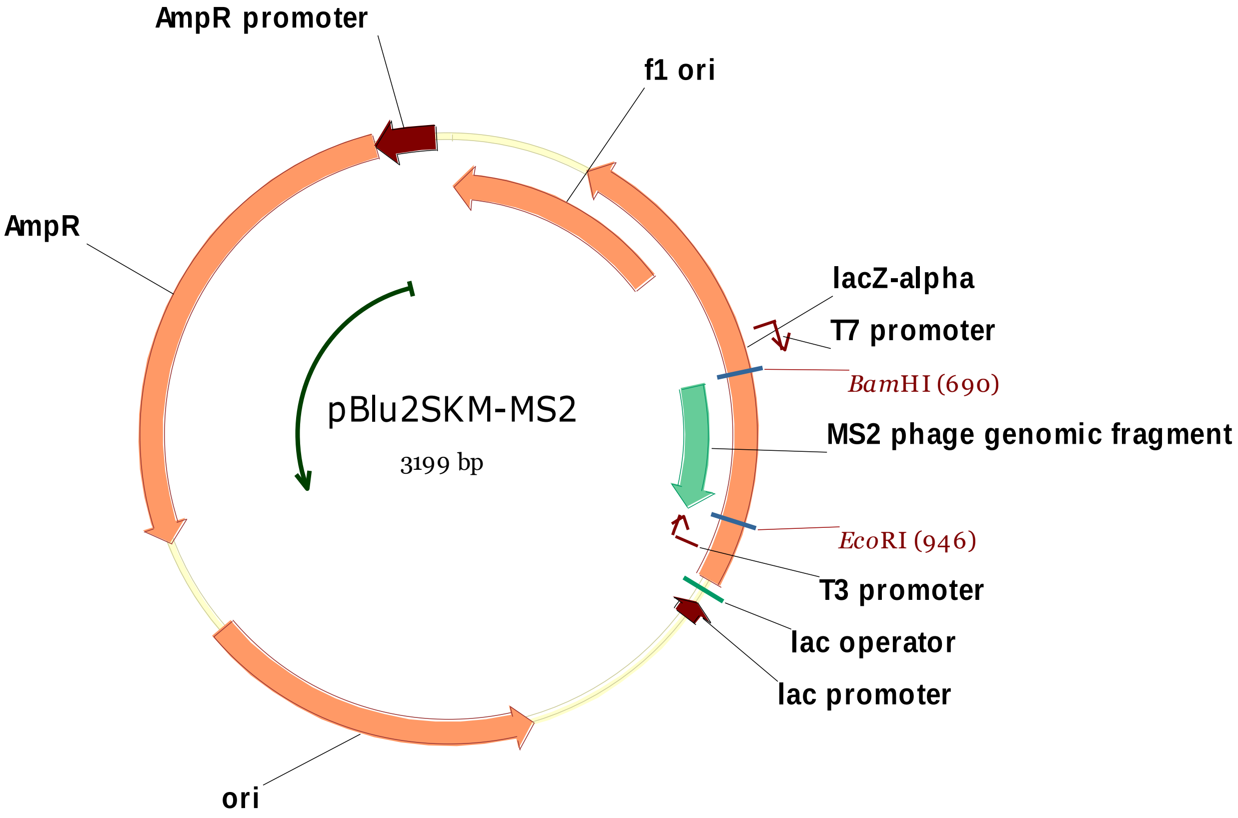

ctaaattgtaagcgttaatattttgttaaaattcgcgttaaatttttgttaaatcagctcattttttaaccaataggccgaaatcggcaaaatcccttataaatc
aaaagaatagaccgagatagggttgagtgttgttccagtttggaacaagagtccactattaaagaacgtggactccaacgtcaaagggcgaaaaaccgtc
tatcagggcgatggcccactacgtgaaccatcaccctaatcaagttttttggggtcgaggtgccgtaaagcactaaatcggaaccctaaagggagcccccg
atttagagcttgacggggaaagccggcgaacgtggcgagaaaggaagggaagaaagcgaaaggagcgggcgctagggcgctggcaagtgtagcggtc
acgctgcgcgtaaccaccacacccgccgcgcttaatgcgccgctacagggcgcgtcccattcgccattcaggctgcgcaactgttgggaagggcgatcggt
gcgggcctcttcgctattacgccagctggcgaaagggggatgtgctgcaaggcgattaagttgggtaacgccagggttttcccagtcacgacgttgtaaaac
gacggccagtgagcgcgcgtaatacgactcactatagggcgaattggagctccaccgcggtggcggccgctctagaactagtggatcctgcctgtaaggag
cctgatatgaatatgtacctccagaaaggggtcggtgctttcatcagacgccggctcaaatccgttggtatagacctgaatgatcaatcgatcaaccagcgt
ctggctcagcagggcagcgtagatggttcgcttgcgacgatagacttatcgtctgcatccgattccatctccgatcgcctggtgtggagttttctcccaccaga
gctatattcatatctcgatcgtatccgctcagaattcgatatcaagcttatcgataccgtcgacctcgagggggggcccggtacccagcttttgttccctttagt
gagggttaattgcgcgcttggcgtaatcatggtcatagctgtttcctgtgtgaaattgttatccgctcacaattccacacaacatacgagccggaagcataaa
gtgtaaagcctggggtgcctaatgagtgagctaactcacattaattgcgttgcgctcactgcccgctttccagtcgggaaacctgtcgtgccagctgcattaa
tgaatcggccaacgcgcggggagaggcggtttgcgtattgggcgctcttccgcttcctcgctcactgactcgctgcgctcggtcgttcggctgcggcgagcgg
tatcagctcactcaaaggcggtaatacggttatccacagaatcaggggataacgcaggaaagaacatgtgagcaaaaggccagcaaaaggccaggaac
cgtaaaaaggccgcgttgctggcgtttttccataggctccgcccccctgacgagcatcacaaaaatcgacgctcaagtcagaggtggcgaaacccgacagg

actataaagataccaggcgtttccccctggaagctccctcgtgcgctctcctgttccgaccctgccgcttaccggatacctgtccgcctttctcccttcgggaag
cgtggcgctttctcatagctcacgctgtaggtatctcagttcggtgtaggtcgttcgctccaagctgggctgtgtgcacgaaccccccgttcagcccgaccgct
gcgccttatccggtaactatcgtcttgagtccaacccggtaagacacgacttatcgccactggcagcagccactggtaacaggattagcagagcgaggtatg
taggcggtgctacagagttcttgaagtggtggcctaactacggctacactagaaggacagtatttggtatctgcgctctgctgaagccagttaccttcggaaa
aagagttggtagctcttgatccggcaaacaaaccaccgctggtagcggtggtttttttgtttgcaagcagcagattacgcgcagaaaaaaaggatctcaag
aagatcctttgatcttttctacggggtctgacgctcagtggaacgaaaactcacgttaagggattttggtcatgagattatcaaaaaggatcttcacctagatc
cttttaaattaaaaatgaagttttaaatcaatctaaagtatatatgagtaaacttggtctgacagttaccaatgcttaatcagtgaggcacctatctcagcgat
ctgtctatttcgttcatccatagttgcctgactccccgtcgtgtagataactacgatacgggagggcttaccatctggccccagtgctgcaatgataccgcgag
acccacgctcaccggctccagatttatcagcaataaaccagccagccggaagggccgagcgcagaagtggtcctgcaactttatccgcctccatccagtct
attaattgttgccgggaagctagagtaagtagttcgccagttaatagtttgcgcaacgttgttgccattgctacaggcatcgtggtgtcacgctcgtcgtttggt
atggcttcattcagctccggttcccaacgatcaaggcgagttacatgatcccccatgttgtgcaaaaaagcggttagctccttcggtcctccgatcgttgtcag
aagtaagttggccgcagtgttatcactcatggttatggcagcactgcataattctcttactgtcatgccatccgtaagatgcttttctgtgactggtgagtactc
aaccaagtcattctgagaatagtgtatgcggcgaccgagttgctcttgcccggcgtcaatacgggataataccgcgccacatagcagaactttaaaagtgct
catcattggaaaacgttcttcggggcgaaaactctcaaggatcttaccgctgttgagatccagttcgatgtaacccactcgtgcacccaactgatcttcagca
tcttttactttcaccagcgtttctgggtgagcaaaaacaggaaggcaaaatgccgcaaaaaagggaataagggcgacacggaaatgttgaatactcatact
cttcctttttcaatattattgaagcatttatcagggttattgtctcatgagcggatacatatttgaatgtatttagaaaaataaacaaataggggttccgcgcac
atttccccgaaaagtgccac

Sequences of cloned reverse transcriptases

Mutations L139P, D200N, T330P are marked by red characters. The sequence of Sto7d is marked by green characters.

RT

MASMTGGQQMGRDPNSLNIEDEHRLHETSKEPDVSLGSTWLSDFPQAWAETGGMGLAVRQAPLIIPLKATST
PVSIKQYPMSQEARLGIKPHIQRLLDQGILVPCQSPWNTPLLPVKKPGTNDYRPVQDLREVNKRVEDIHPTVPNP
YNLLSGLPPSHQWYTVLDLKDAFFCLRLHPTSQPLFAFEWRDPEMGISGQLTWTRLPQGFKNSPTLFDEALHRDL
ADFRIQHPDLILLQYVDDLLLAATSELDCQQGTRALLQTLGNLGYRASAKKAQICQKQVKYLGYLLKEGQRWLTEA
RKETVMGQLTPKTPRQLREFLGTAGFCRLWIPGFAEMAAPLYPLTKTGTLFNWGPDQQKAYQEIKQALLTAPAL
GLPDLTKPFELFVDEKQGYAKGVLTQKLGPWRRPVAYLSKKLDPVAAGWPPCLRMVAAIAVLTKDAGKLTMGQ
PLVILAPHAVEALVKQPPDRWLSNARMTHYQALLLDTDRVQFGPVVALNPATLLPLPEEGLQHNCLDQAKLAAA
LEHHHHHH

RT mut

MASMTGGQQMGRDPNSLNIEDEHRLHETSKEPDVSLGSTWLSDFPQAWAETGGMGLAVRQAPLIIPLKATST
PVSIKQYPMSQEARLGIKPHIQRLLDQGILVPCQSPWNTPLLPVKKPGTNDYRPVQDLREVNKRVEDIHPTVPNP
YNLLSGPPPSHQWYTVLDLKDAFFCLRLHPTSQPLFAFEWRDPEMGISGQLTWTRLPQGFKNSPTLFNEALHRD
LADFRIQHPDLILLQYVDDLLLAATSELDCQQGTRALLQTLGNLGYRASAKKAQICQKQVKYLGYLLKEGQRWLTE
ARKETVMGQLTPKTPRQLREFLGTAGFCRLWIPGFAEMAAPLYPLTKPGTLFNWGPDQQKAYQEIKQALLTAPA
LGLPDLTKPFELFVDEKQGYAKGVLTQKLGPWRRPVAYLSKKLDPVAAGWPPCLRMVAAIAVLTKDAGKLTMG
QPLVILAPHAVEALVKQPPDRWLSNARMTHYQALLLDTDRVQFGPVVALNPATLLPLPEEGLQHNCLDQAKLAA
ALEHHHHHH

RT-Sto

MASMTGGQQMGRDPNSLNIEDEHRLHETSKEPDVSLGSTWLSDFPQAWAETGGMGLAVRQAPLIIPLKATST
PVSIKQYPMSQEARLGIKPHIQRLLDQGILVPCQSPWNTPLLPVKKPGTNDYRPVQDLREVNKRVEDIHPTVPNP
YNLLSGLPPSHQWYTVLDLKDAFFCLRLHPTSQPLFAFEWRDPEMGISGQLTWTRLPQGFKNSPTLFDEALHRDL
ADFRIQHPDLILLQYVDDLLLAATSELDCQQGTRALLQTLGNLGYRASAKKAQICQKQVKYLGYLLKEGQRWLTEA
RKETVMGQLTPKTPRQLREFLGTAGFCRLWIPGFAEMAAPLYPLTKTGTLFNWGPDQQKAYQEIKQALLTAPAL
GLPDLTKPFELFVDEKQGYAKGVLTQKLGPWRRPVAYLSKKLDPVAAGWPPCLRMVAAIAVLTKDAGKLTMGQ
PLVILAPHAVEALVKQPPDRWLSNARMTHYQALLLDTDRVQFGPVVALNPATLLPLPEEGLQHNCLDGTGGGG
VTVKFKYKGEEKEVDISKIKKVWRVGKMISFTYDDNGKTGRGAVSEKDAPKELLQMLEKSGKKAAALEHHHHHH

RT-Sto mut

MASMTGGQQMGRDPNSLNIEDEHRLHETSKEPDVSLGSTWLSDFPQAWAETGGMGLAVRQAPLIIPLKATST
PVSIKQYPMSQEARLGIKPHIQRLLDQGILVPCQSPWNTPLLPVKKPGTNDYRPVQDLREVNKRVEDIHPTVPNP
YNLLSGPPPSHQWYTVLDLKDAFFCLRLHPTSQPLFAFEWRDPEMGISGQLTWTRLPQGFKNSPTLFNEALHRD
LADFRIQHPDLILLQYVDDLLLAATSELDCQQGTRALLQTLGNLGYRASAKKAQICQKQVKYLGYLLKEGQRWLTE
ARKETVMGQLTPKTPRQLREFLGTAGFCRLWIPGFAEMAAPLYPLTKPGTLFNWGPDQQKAYQEIKQALLTAPA
LGLPDLTKPFELFVDEKQGYAKGVLTQKLGPWRRPVAYLSKKLDPVAAGWPPCLRMVAAIAVLTKDAGKLTMG
QPLVILAPHAVEALVKQPPDRWLSNARMTHYQALLLDTDRVQFGPVVALNPATLLPLPEEGLQHNCLDGTGGG
GVTVKFKYKGEEKEVDISKIKKVWRVGKMISFTYDDNGKTGRGAVSEKDAPKELLQMLEKSGKKAAALEHHHHH
H

a)

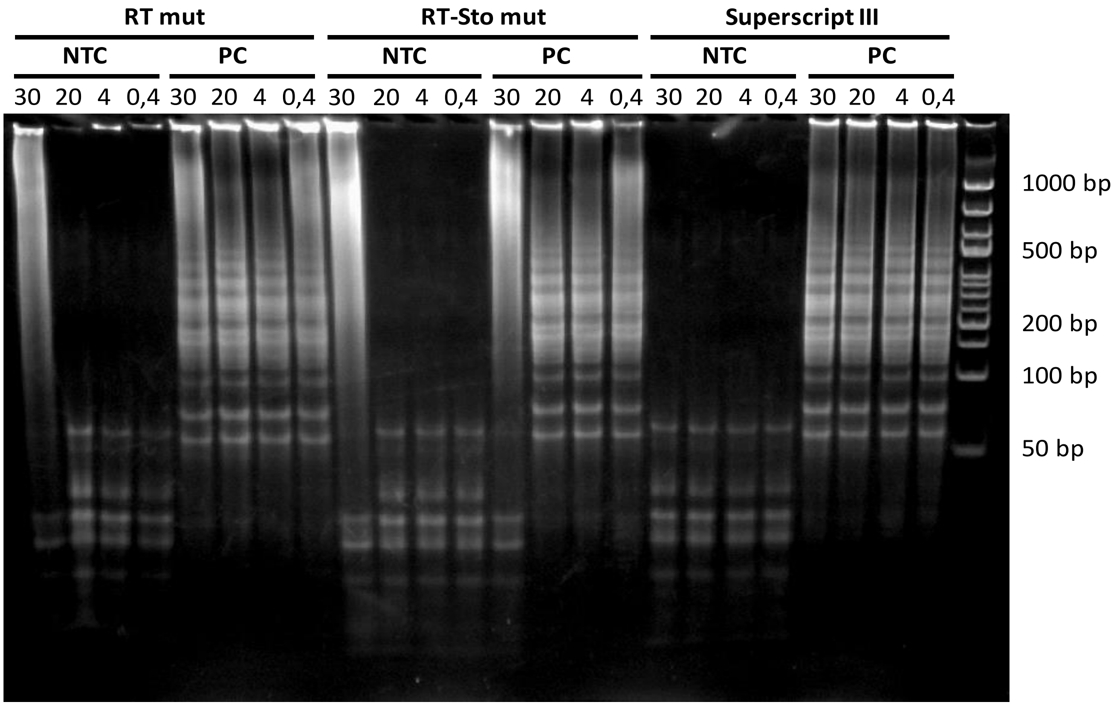
RT mut
RT-Sto mut
Superscript III
NTC
PC
NTC
PC
NTC
PC
30 20 4 0,4 30 20 4 0,4 30 20 4 0,4 30 20 4 0,4 30 20 4 0,4 30 20 4 0,4
1000 bp
500 bp
200 bp
100 bp
50 bp

b)

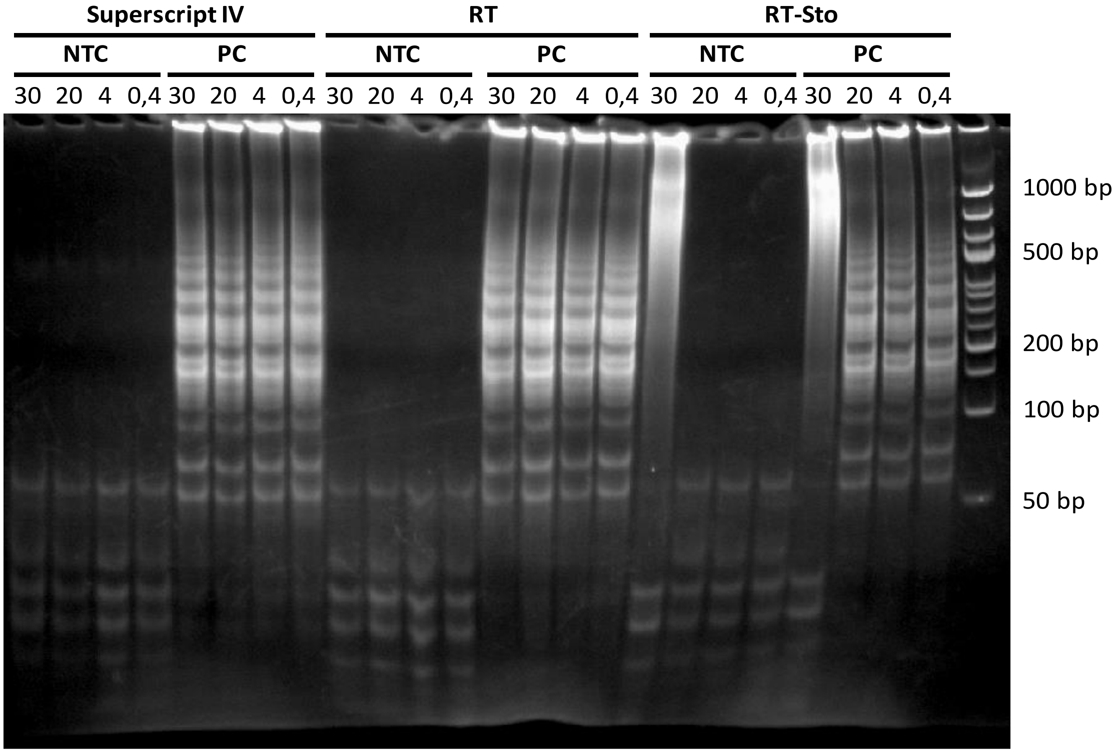
Superscript IV
RT
RT-Sto
NTC
PC
NTC
PC
NTC
PC
30 20 4 0,4 30 20 4 0,4 30 20 4 0,4 30 20 4 0,4 30 20 4 0,4 30 20 4 0,4
1000 bp
500 bp
200 bp
100 bp
50 bp

c)

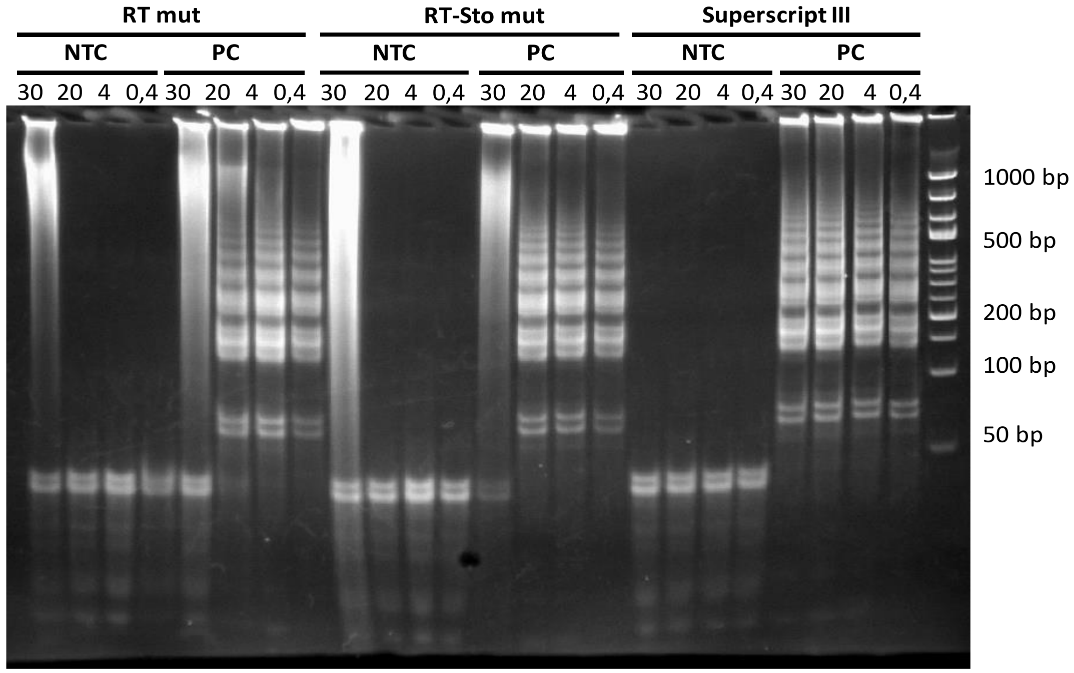

d)

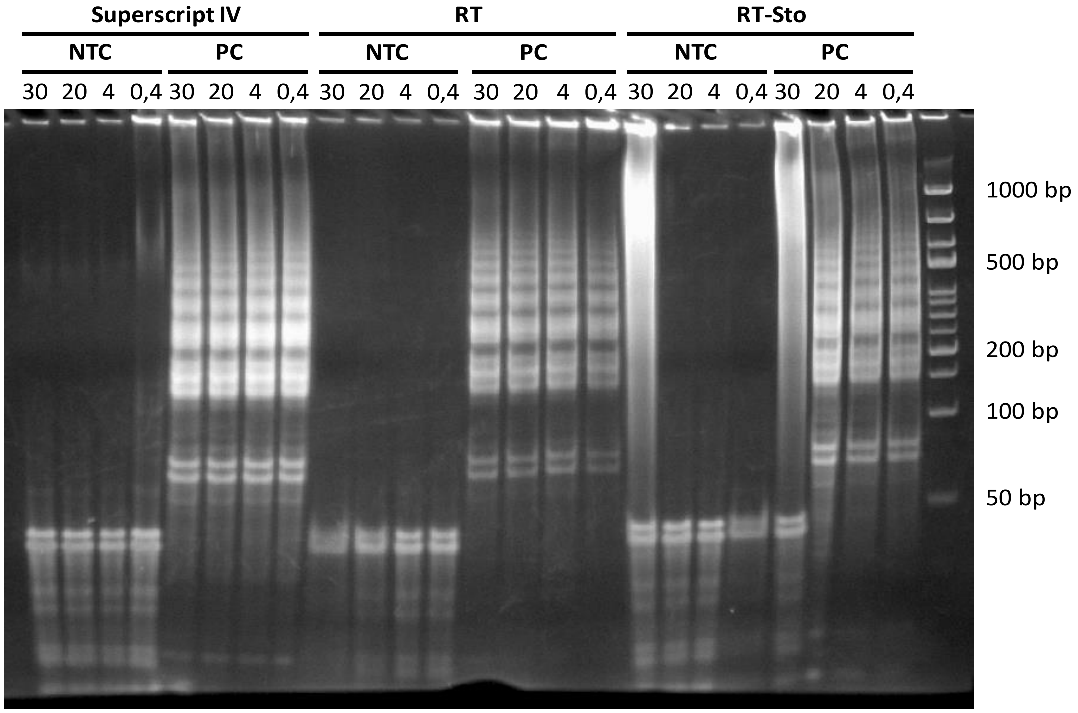

Supplementary Figure S1. RT-LAMP with various amounts of RTs and primers for MS2 (**a, b**) and SARS-CoV-2 (**c, d**). The temperature of the reverse transcription step was 42 °C. Each enzyme is marked by the color specified in the legend. SARS-CoV-2 RNA fragment and MS2 genomic RNA were added in reactions to a final concentration of 10^3 copies per reaction. NTC – no template control, PC – positive control, RNA template was added to the reactions. The results are shown for 30, 20, 4, 0.4 units of enzyme per reaction. Each experiment was triplicated.
